# Metal-Organic Frameworks: A Potential Platform for Enzyme Immobilization and Related Applications

**DOI:** 10.3389/fbioe.2020.00695

**Published:** 2020-06-30

**Authors:** Huan Xia, Na Li, Xue Zhong, Yanbin Jiang

**Affiliations:** School of Chemistry and Chemical Engineering, Guangdong Provincial Key Lab of Green Chemical Product Technology, South China University of Technology, Guangzhou, China

**Keywords:** enzyme immobilization, metal-organic frameworks, synthetic strategy, catalysis, bio-transformation, sensing

## Abstract

Enzymes, as natural catalysts with remarkable catalytic activity and high region-selectivities, hold great promise in industrial catalysis. However, applications of enzymatic transformation are hampered by the fragility of enzymes in harsh conditions. Recently, metal–organic frameworks (MOFs), due to their high stability and available structural properties, have emerged as a promising platform for enzyme immobilization. Synthetic strategies of enzyme-MOF composites mainly including surface immobilization, covalent linkage, pore entrapment and *in situ* synthesis. Compared with free enzymes, most immobilized enzymes exhibit enhanced resistance against solvents and high temperatures. Besides, MOFs serving as matrixes for enzyme immobilization show extraordinary superiority in many aspects compared with other supporting materials. The advantages of using MOFs to support enzymes are discussed. To obtain a high enzyme loading capacity and to reduce the diffusion resistance of reactants and products during the reaction, the mesoporous MOFs have been designed and constructed. This review also covers the applications of enzyme-MOF composites in bio-sensing and detection, bio-catalysis, and cancer therapy, which is concerned with interdisciplinary nano-chemistry, material science and medical chemistry. Finally, some perspectives on reservation or enhancement of bio-catalytic activity of enzyme-MOF composites and the future of enzyme immobilization strategies are discussed.

## Introduction

Enzymes are undisputedly one of most significant macromolecular biological catalysts, which are widely used in fields such as fine chemicals and drug synthesis, sensing, energy and food processing. Enzymes excel in some highly selective (region-, stereo-, chemo-) transformation processes with high efficiency and turnover numbers than artificial catalysts under environmentally benign conditions (Schmid et al., 2001; van Dongen et al., 2014). However, the industrial application of enzymes suffers from their fragile nature such as poor operational, thermal, chemical, and storage stability (Strohmeier et al., 2011; Bornscheuer et al., 2012; Zhou and Hartmann, 2013). Also, the residual enzyme may serve as a contaminant in product mixture during the production process, which requires expensive separation and purification processes. Although advances in gene and protein engineering could tune the nature of enzymes to improve substrate recognition, catalytic efficiency and operational stability, the immobilization of enzymes on solid supports by chemical modification is the most common way to improve the practical performance of enzymes (Franssen et al., 2013; Huang et al., 2020).

Enzyme immobilization can improve enzyme stability, recyclability and recovery in a low-cost way by confining enzyme in a specific space with preserved enzymatic activity (Tosa et al., 1966; Li et al., 2020). Solid supports show that the porous characteristics emerge as optional platforms for enzyme immobilization. These materials include, but are not restricted to, bulk materials (Sheldon, 2007), particles (Ansari and Husain, 2012), hydrogels (Lee et al., 2007), graphene oxide (Pavlidis et al., 2014), carbon nanotubes (Feng and Ji, 2011), mesoporous silica (Hudson et al., 2008; Hartmann and Jung, 2010; Magner, 2013), and polymers (Woodward and Kaufman, 1996; Veronese, 2001). The immobilization of enzymes on these materials may inevitably lead to a low enzyme loading, enzyme denaturation, or restricted mass transfer. Therefore, to maximize the activity and stability of the immobilized enzyme, it is important to recognize the matrix and strategies, since the physical and chemical properties of enzymes may change during the immobilization process (Mohamad et al., 2015).

Metal-Organic Frameworks (MOFs) are composed of metal nodes and organic ligands connected through coordination bonds, which shows great potential in various applications (Kleist et al., 2010; Cho et al., 2012; Ding et al., 2019; Masoomi et al., 2019; Shi et al., 2019; Wang et al., 2019; Yilmaz et al., 2019; Abanades Lazaro et al., 2020; Bonneau et al., 2020; Cruz-Navarro et al., 2020). Given that the topology and property of MOFs could be tuned for their multiple metal nodes and ligands, interactions such as hydrogen bonding, van der Waals forces, covalence, and coordinative bonding between MOFs support and enzyme are possible (Majewski et al., 2017). Besides, the very high surface, pore volume, and easy modification make MOFs a potential supporting matrix for enzyme immobilization (Morris et al., 2014; Mehta et al., 2016; Doonan et al., 2017; Gkaniatsou et al., 2017; Lian et al., 2017). Last but not the least, the ordered crystal structure and the high tunability of MOFs can provide for uniform loading with less leaching, and enzyme’s microenvironment, which protects enzymes from harsh thermal and chemical conditions (Drout et al., 2019). This paper reviews the current strategies for enzyme immobilization on MOF materials, and the merits of enzyme immobilized with MOFs are discussed with an emphasis on the application of enzyme-MOF composites.

## Strategies for Enzyme Immobilized by MOFs

**FIGURE 1:**
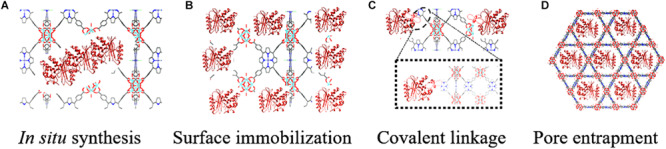
Schematic representation of different immobilization methods for enzymes. **(A)** *In situ* synthesis. **(B)** Surface immobilization. **(C)** Covalent linkage. **(D)** Pore entrapment.

Figure 1 shows the schematic representation of different immobilization methods for enzymes. Typically, there are mainly two ways to prepare enzyme-MOF composites in terms of how enzymes bind to the supports. One is that enzymes are immobilized into the MOF during its formation process in a *de novo*/*in situ* approach. The other is a post-synthetic method where enzymes are introduced into the pre-existing MOF, including approaches of surface immobilization, covalent linkage and pore entrapment. Each route ensures the immobilization conditions do not exceed the denaturation ranges of enzymes. Although MOFs can endow enzymes with remarkable stabilities against harsh conditions, factors that MOFs may have on the immobilized enzyme such as substrate diffusion, activity amplification effects, and selectivity should also be considered. Table 1 summarizes the preparation and application of enzyme-MOF composites.

**TABLE 1:** Examples of preparation and application of the enzyme-MOF composites.

**Method**	**MOF**	**Enzyme**	**Application**	**References**
*In situ* synthesis	ZIFs	Cytochrome *c*, horseradish peroxidase, lipase, etc.	Bio-sensing	Lyu et al., 2014; Chulkaivalsucharit et al., 2015; Wu et al., 2017; Zhang et al., 2017
*In situ* synthesis	ZIF-8, HKUST-1, etc.	Horseradish peroxidase, trypsin, urease, etc.	Proof of concept	Liang et al., 2015b; Liang et al., 2016; Tadepalli et al., 2018
*In situ* synthesis	ZIFs	Catalase	Bio-sensing	Shieh et al., 2015; Liang et al., 2019
*In situ* synthesis	ZIF-8	Lipase, β-galactosidase, glucose oxidase, etc.	Bio-sensing	Hou et al., 2015; Huo et al., 2015; Liang et al., 2015a; Wu et al., 2015a, b; Wang Y. et al., 2016; Wang et al., 2017; Mohammad et al., 2019
*In situ* synthesis	ZIF-8	Glucose oxidase, horseradish peroxidase	Proof of concept, bio-catalysis	Cheng et al., 2019
*In situ* synthesis	ZIF-8	Lipase	Kinetic resolution of (*R, S*)-2-octanol	He et al., 2016
*In situ* synthesis	MIL-88A	Dehydrogenase, horseradish peroxidase, acetylcholinesterase	Proof of concept	Jeong et al., 2015
*In situ* synthesis	Fe/Cu-MOF	Laccase, lipase, cytochrome *c*, dehydrogenase, etc.	Bio-catalysis	Gascón et al., 2017a; Li et al., 2017; Gascón et al., 2018; Xia et al., 2019
*In situ* synthesis	La/Fe/Zr-MOF	Acetylcholinesterase	Bio-sensing	Dong et al., 2018
*In situ* synthesis	Al/Mg-MOF	β-glucosidase, laccase	Proof of concept	Gascón et al., 2017b
Surface immobilization	UiO-66, MIL-53	Lipase	Warfarin synthesis	Liu et al., 2015
Surface immobilization	HKUST-1	Lipase	Esterification	Cao Y. et al., 2016
Surface immobilization	Zr-MOF	Laccase	Proof of concept	Pang et al., 2016
Surface immobilization	Cu-MOF, ZIFs	Trypsin, tyrosinase, etc.	Bio-sensing	Ma et al., 2013; Wang et al., 2015; Zhao et al., 2015; Lu et al., 2016
Surface immobilization	MOF-545	Glucose oxidase	Bio-sensing	Zhong et al., 2020
Surface immobilization	MIL-100, HKUST-1	Lipase, glucose oxidase, etc.	Bio-catalysis and biosensing	Patra et al., 2015; Nobakht et al., 2018
Surface immobilization	Cu-MOF	Microperoxidase-11	Bio-catalysis	Pisklak et al., 2006
Surface immobilization	CYCU-4, UiO-66	Trypsin	BSA digestion	Liu et al., 2013; Liu et al., 2014
Covalent linkage	UiO-66-NH_2_	Hydrolase	Asymmetric hydrolysis	Cao S. L. et al., 2016
Covalent linkage	IRMOF-3	Protein, lipase	Transesterification	Jung et al., 2011
Covalent linkage	MIL-101-NH_2_	Hemin	Bio-sensing	Qin et al., 2013
Covalent linkage	MIL-125	Hemoglobin	Proof of concept	Wang W. et al., 2016
Covalent linkage	ZIF-8, MIL-88B-NH_2_	Trypsin	Proteolysis	Shih et al., 2012; Wen et al., 2016
Pore entrapment	Tb-mesoMOF	Myoglobin, microperoxidase-11	Bio-catalysis	Lykourinou et al., 2011; Chen et al., 2012a
Pore entrapment	IRMOF-74, etc.	Myoglobin, protein	Proof of concept	Deng et al., 2012
Pore entrapment	NU-1003, PCN-128y	Anhydrolase	Detoxifying DFP and Soman	Li et al., 2016b, c
Pore entrapment	PCN-333	Microperoxidase-11, cytochrome *c*, horseradish peroxidase	Bio-catalysis	Feng et al., 2015
Pore entrapment	PCN-888	Glucose oxidase, horseradish peroxidase	Proof of concept	Lian et al., 2016
Pore entrapment	Tb-TATB	Cytochrome *c*	Mechanism comprehension	Chen et al., 2012b

We start with the recently popularized examples of the *in situ* encapsulation method, which is also known as co-precipitation or mineralization. Then, post-synthetic approaches such as surface immobilization, covalent linkage, and pore entrapment are discussed in sequence.

### *In situ* Synthesis

Mild operating conditions are the key for *in situ* enzyme-MOFs synthesis where enzymes and MOF precursors (metal ions and organic ligands) are mixed with the most common aqueous solution. This method allows for the nucleation and growth of MOF simultaneously, and the size of the gust molecule can be larger than the pore size of MOFs, resulting in enzyme embedded MOF crystals (Figure 1A).

**FIGURE 2:**
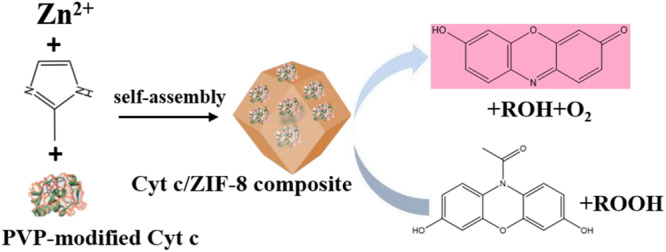
Preparation of the Cyt *c*-embedded ZIF-8. A schematic showing the synthesis of the Cyt *c*/ZIF-8 composite and showing the detection of organic peroxides.

Zeolitic imidazolate framework (ZIF) is the first to be used to immobilize enzyme *in situ* for its extremely mild synthetic conditions. Lyu et al. (2014) initially reported the cytochrome *c* (Cyt *c*) embedded in a ZIF-8 by mixing zinc nitrate hexahydrate, 2-methylimidazole, and polyvinylpyrrolidone (PVP) modified Cyt *c* in methanol (Figure 2). Modern characterization techniques confirmed that embedded Cyt *c* did not affect the morphology and the crystalline structure of ZIF-8. The enzymatic activity of immobilized Cyt *c* was assayed by using 2,2′-azinobis(2-ethylbenzthiazoline)-6-sulronate (ABTS) and H_2_O_2_ as substrates in potassium phosphate buffer. The immobilized Cyt *c* displayed a 10-fold enhanced apparent activity than free Cyt *c*, which could be attributed to the conformational changes of Cyt *c* incubated in the methanol, resulting in an exposed heme group. This phenomenon that the conformational changes of Cyt *c* resulted in a favorable catalytic performance was also observed in the research of Wu et al. (2020). The enzymatic Michaelis-Menten kinetics analysis suggested that the immobilized Cyt *c* exhibited the decreased *K*_*m*_ (Michaelis-Menten constant) and the increased *V*_*max*_ (maximum rate of reaction), compared with the free enzyme, indicating a higher affinity toward substrates for the immobilized Cyt *c*. Then, the Cyt *c*/ZIF-8 was applied for the detection of H_2_O_2_, methyl ethyl ketone peroxide, and *tert*-butyl hydroperoxide, using Amplex Red (10-acetyl-3,7-dihydroxyphenoxazine) as the substrate. The results also demonstrated that Cyt *c*/ZIF-8 showed good linearity and sensitivity than free Cyt *c*.

Based on this approach, Wu et al. (2015a) reported a bi-enzymatic system, where glucose oxidase (GOx) and horseradish peroxidase (HRP) were encapsulated *in situ* during ZIF-8 formation. The synthesized GOx&HRP@ZIF-8 composites were performed to display the effective enzymatic cascade reactions, and served as the glucose sensor, resulting in the detection limit of 0.5 μM. Notably, the multiple enzyme-embedded ZIF-8 nanocrystals showed high selectivity toward glucose, even in the higher concentration of various interfering compounds such as fructose, mannose, galactose, maltose, lactose and albumin. Besides, the immobilized enzymes showed greatly enhanced stability against proteolysis and chelating, owing to the structural rigidity and confinement of MOF scaffolds.

**FIGURE 3:**
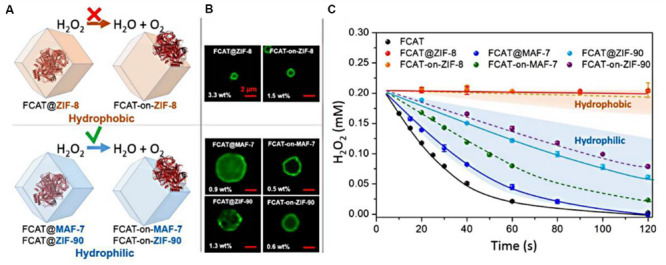
**(A)** Schematic representations of the different FCAT/ZIF bio-composites formed by encapsulation of enzyme molecules via biomimetic mineralization or surface adsorption within/on hydrophobic (orange) or hydrophilic (blue) frameworks. **(B)** Confocal laser scanning micrographs showing fluorescence of different FCAT/ZIF bio-composites. **(C)** Catalytic activity of FCAT and different FCAT/ZIF composites. The assay was performed with FCAT concentration of 20 nM and H_2_O_2_ concentration of 0.20 mM. Reproduced from Liang et al. (2019) with permission from the American Chemical Society, copyright 2019.

Liang et al. (2019) developed this *in situ* approach to encapsulate enzymes within a series of ZIFs, such as MAF-7, ZIF-90, and ZIF-8. The particular catalase (CAT), a kind of iron-heme enzyme that catalyzes the decomposition of hydrogen peroxide to water and oxygen, was chosen as a model bio-catalyst to investigate whether the hydrophilicity of matrixes was related to their biological compatibility. The hydrophilic MAF-7 (links: 3-methyl-1,2,4-triazolate) and ZIF-90 (links: 2-imidazolate carboxaldehyde), as well as the hydrophobic ZIF-8 were used to encapsulate catalase to synthesize enzyme-ZIF composites. The enzymatic assays indicated that the FCAT@MAF-7 showed the approximate activity to that of free FCAT, which exhibited the optimal catalytic performance. Whereas, FCAT that physically adsorbed on the surface of ZIF-8 or encapsulated in the ZIF-8 showed the negligible catalytic activity in H_2_O_2_ decomposition (Figures 3A,C). Fluorescein isothiocyanate tagged with CAT (FCAT) was performed to investigate the spatial distribution of enzymes within samples using confocal laser scanning microscopy. FCAT molecules were more homogeneously distributed throughout FCAT@MAF-7 and FCAT@ZIF-90 than in FCAT@ZIF-8 (Figure 3B). These results suggested that the rational design of hydrophilic and hydrophobic interactions between enzymes and matrixes is essential for the efficiency and stabilization of biomolecules.

**FIGURE 4:**
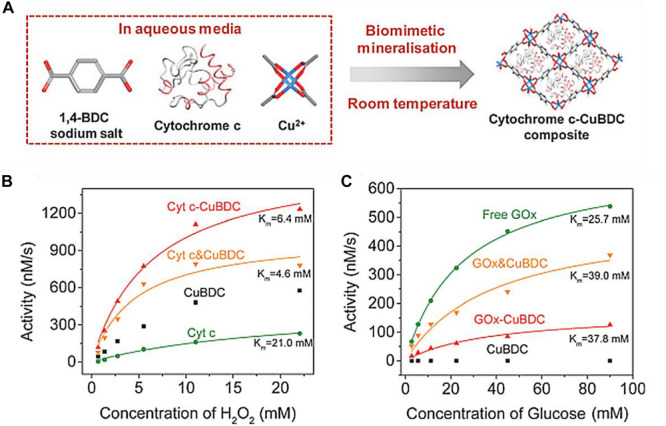
**(A)** Schematic illustration of preparing enzyme-CuBDC composites in one-pot and an *in situ* approach by biomimetic mineralization. **(B)** The kinetic analysis of CuBDC, free Cyt *c*, Cyt *c*-CuDBC, and Cyt *c*&CuBDC. **(C)** The kinetic analysis of CuBDC, free GOx, GOx-CuBDC, and GOx&CuBDC. Reproduced from Li et al. (2017) with permission from the Royal Society of Chemistry, copyright 2017.

The further applications of this *in situ* synthetic approach toward different enzymes and MOFs materials have also been reported (Jeong et al., 2015; Li et al., 2017; Gascón et al., 2017a; Gascón et al., 2018; Feng et al., 2019; Chen et al., 2019; Xia et al., 2019; Chen et al., 2020). For example, Li et al. (2017) converted 1, 4-benzenedicarboxylic acid (H_2_BDC) to its sodium salt form by sodium hydroxide, to keep the BDC ligand soluble in aqueous solution. Then Cyt *c* was mixed with Cu ion and soluble BDC to synthesize the Cyt *c*-CuBDC composites (Figure 4A). Spatial distribution analysis of immobilized enzyme demonstrated the enzyme was incorporated into the skeleton of the CuBDC. Owing to the intrinsic peroxidase-like activity of CuBDC and Cu ion activation effect, the Cyt *c*-CuBDC composites achieved an approximate 12-fold catalytic efficiency compared to free Cyt *c*. The activity enhancement was also observed for the Cyt *c*&CuBDC prepared by physical mixing, due to the activity superposition of Cyt *c* and CuBDC by additive effect (Figure 4B). However, this activity enhancement was not observed for GOx. The varying degree of activity loss was observed for GOx-CuBDC and GOx&CuBDC composites (Figure 4C).

Though these preliminary researches are promising, the encapsulation of enzyme into MOFs by this *in situ* approach largely relies on the synthetic conditions, which should be simple and biocompatible. Therefore, only a few MOFs are the candidates for this immobilization method. Moreover, spatial localization and dispersion of enzyme as well as the control of MOF particles and MOF coating thickness needs to be investigated and optimized for further bio-catalytic applications.

### Surface Immobilization

Surface immobilization is simple, low-cost, and most commonly used for protein anchored on various solid supports. Enzymes immobilized on MOF surfaces are mainly through weak interactions such as van der Waals forces, π-π interaction, electrostatic interaction, or hydrogen bonding (Figure 1B). The turbulence of pH and salt concentrations could have a great effect on the interactions between enzymes and supports. This immobilization process is reversible, thus enzyme leaking from the support could be observed if the synthetic conditions are not chosen properly. An extensive of MOFs can serve as the matrixes for enzyme immobilization, for there is no strict requirement for particular functional groups or pore size of MOFs.

Earlier, Balkus et al. employed a nano-crystalline Cu-MOF to immobilize microperoxidase-11 (MP-11) by physical adsorption at room temperature (Pisklak et al., 2006). Meanwhile, five different mesoporous benzene silica (MBS) molecular sieve samples were also performed to immobilized MP-11 as control groups. MP-11 was immobilized in all these MBS matrixes through van der Waals interaction between the MP-11 and the pore walls of MBS supports. MBS materials that contained pendent amine group would facilitate the uptake of the MP-11. The enzymatic activity assays suggested that all of these MP-11@MBS samples showed a much lower reactant conversion than the free MP-11, which could be attributed to the limitation of MP-11 imposed by the confined space of MBS matrixes. Notably, the MP-11@Cu-MOF showed a much higher reactant conversion (63 ± 5.1%) than that of free MP-11 (6 ± 5.2%). The results suggested that MOF exceled in the consolidation of immobilized enzyme than the MBS materials.

**FIGURE 5:**
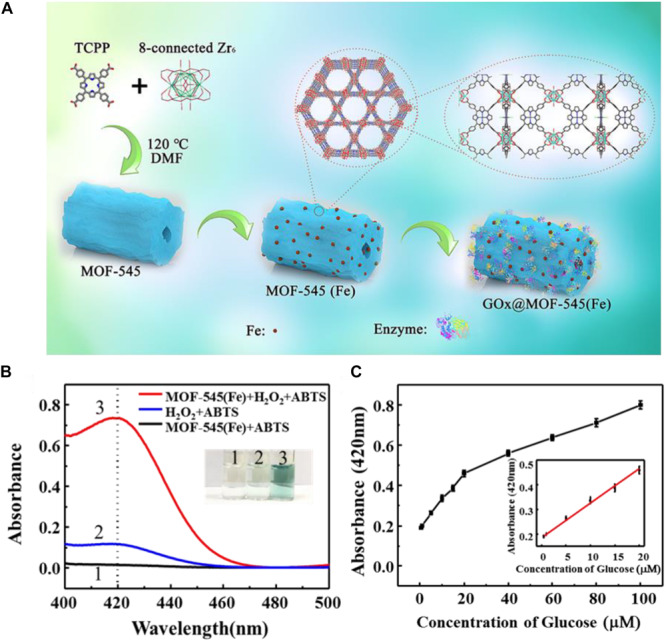
**(A)** Schematic illustration of the preparation of GOx@MOF-545(Fe) composites through a surface adsorption approach and cascade reaction based on GOx@MOF-545(Fe). **(B)** UV-Vis absorption spectra of different reaction systems. **(C)** Detection of glucose by GOx@MOF-545(Fe) with the increase of glucose concentration from 0.5 to 100 μM and 0.5–20 μM (inset). Reproduced from Zhong et al. (2020) with permission from Elsevier, copyright 2020.

Recently, using the same approach, Zhong et al. (2020) immobilized GOx on a biomimetic Zr-based porphyrin MOF matrix [MOF-545(Fe))], which was constructed using highly stable Zr cluster as nodes and meso-tetra(4-carboxyphenyl) porphine as ligand and followed by introducing ferric ion into the center of the porphyrin structure, resulting in the peroxidase-like activity (Figure 5A). In this system, MOF-545(Fe) not only worked as the matrix for GOx immobilization, but also contributed its peroxidase-like activity to co-operate with GOx for the cascade reactions. The peroxidase-like activity of MOF-545(Fe) was assayed, and ABTS served as a color development reagent with its absorbance at 420 nm tested by a UV-vis spectrophotometer. Only when the reaction system with MOF-545(Fe), ABTS and H_2_O_2_, a significant absorbance at 420 nm was observed, and the mixed solution turned to clear green (Figure 5B). The GOx@MOF-545(Fe) was applied for glucose detection with the one-step cascade reaction. A limit of detection (LOD) of 0.28 μM was achieved with a good linear relationship in the range of 0.5–20 μM (Figure 5C). Such a great LOD value could be ascribed to the hierarchical porous of the MOF-545(Fe), which facilitated the substrate mass transfer in the glucose detection reactions.

Liu et al. successfully employed a Cu-BTC (BTC: 1,3,5-benzenertricarboxylate) based hierarchically porous MOF matrix to immobilize the *Bacillus subtilis* lipase (BSL2) (Cao Y. et al., 2016). In this research, the BSL2 was modified by the non-ionic surfactant (dioleyl-N-D-glucona-L-glutamate) to synthesize BSL2-surfactant complexes, which were further adsorbed to the hierarchically porous Cu-BTC, followed by washing with isooctane and lyophilization. The BSL2@Cu-BTC composites obtained were applied for the esterification reaction between benzyl alcohol and lauric acid. Notably, a 17-fold initial rate was achieved for immobilized BSL2 compared with the free one, which suggested that BSL2 could be fully dispersed on the inner surface of the hierarchically porous Cu-BTC, refraining from aggregation or other inactivation situations (Garcia-Galan et al., 2011). In addition, the novel BSL2@Cu-BTC composites were observed to show good operational stability. These results suggested that the hierarchically porous MOF materials held potentials for applications of biological hybrid materials in catalysis. Other matrixes such as Zr/Al/Fe/Cr-based MOFs have also been widely used to immobilize different enzymes through the surface immobilization technique, which is well documented by Liang and coworkers (Liang et al., 2020).

### Covalent Linkage

Although a few enzymes have been successfully anchored on the surface of MOFs by a physical technique, adsorbed enzymes demonstrate poor stability in the reaction media due to weak interactions between protein and matrix (Cui et al., 2018). Generally, the covalent linkage is the conventional technique for enzyme immobilization. Typically, the abundance of amino groups on an enzyme’s surface can form peptide bonds with carboxyl functionalized MOFs. The covalently linked enzymes usually reveal remarkably improved stability after repeated uses when compared to their non-covalently linked counterparts. In this strategy, several MOFs could be modified with functional groups, e.g., amino, carboxyl and hydroxyl, to serve as immobilization matrixes (Figure 1C).

**FIGURE 6:**
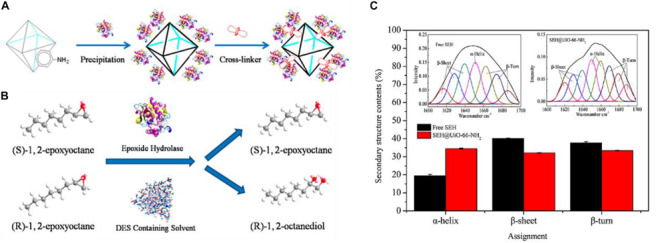
**(A)** Schematic illustration of the synthesis of the immobilization of SEH onto UiO-66-NH_2_. **(B)** Schematic illustration of epoxide hydrolase-catalyzed asymmetric hydrolysis of (*R*/*S*)-1,2-epoxyocatane to chiral (*R*)-1,2-octanediol. **(C)** Amide I fitting results of free SEH and immobilized SEH via FTIR analysis. Reproduced from Cao S. L. et al. (2016) with permission from the American Chemical Society, copyright 2016.

In a pioneering work, Lou et al. immobilized the soybean epoxide hydrolase (SEH) onto the prepared Zr-based MOF matrix, UiO-66-NH_2_ (links: 2-aminoterephthalic acid) (Cao S. L. et al., 2016). Glutaraldehyde was used as a cross-linker since it could easily bind to reactive groups of the enzyme and cross-link them on the MOF surface (Figure 6A). A high SEH loading (87.3 mg/g) with enzyme activity recovery (88.0%) was achieved. The synthetic enzyme-MOF composites were used for the asymmetric hydrolysis of 1,2-epoxyoctane to (*R*)-1,2-octanediol in a novel deep eutectic solvent (Figure 6B). The immobilized SEH not only displayed good long-term stability with 97.5% of its initial activity after being stored at 4°C for 4 weeks, but also remarkably surpassed the free SEH as to pH stability, thermostability, and resistance against organic solvents. These outperformances of immobilized enzyme compared to free enzyme could be attributed to the increase of structural rigidity of the immobilized SEH, which was demonstrated by the secondary structural analysis of protein (Figure 6C). Moreover, the kinetic analysis suggested that SEH@UiO-66-NH_2_ achieved a decreased *K*_*m*_ value and the increased *V_*max*_/K_*m*_* value compared with free SEH, indicating the improved enzyme-substrate affinity and catalytic efficiency for SEH@UiO-66-NH_2_. A possible explanation for this observation could be the facilitation for the access of substrates to enzyme’s active site due to the changes of the three-dimensional structure of SEH upon immobilization. This covalent binding method was also employed to immobilize other enzymes such as trypsin and lipase by different functional groups of modified MOF matrixes (Table 1).

### Pore Entrapment

The pore entrapment or cage encapsulation approach refers to the encapsulation of enzymes into the pores or cages of mesoporous MOFs by diffusion (Figure 1D). This approach has been extensively employed to entrap small-sized objects, such as inorganic nanoparticles or molecular complexes into the pores of MOFs (Horcajada et al., 2012). In contrast to other immobilization techniques, this method sheltered enzymes from deactivation in harsh conditions (e.g., organic solvents, extremely acidic or basic solutions, high temperatures) to a great degree by isolating protein molecules in the pores or cages of MOFs. In addition, the entrapment of enzymes in 3D microenvironments of mesoporous MOFs allowed enzymes to have easier access to substrates and enzyme aggregation could be alleviated with minimized protein unfolding (Majewski et al., 2017). It is worth noting that the mesoporous MOFs used to entrap enzymes have the following advantages over microporous counterparts. First, a very high enzyme loading could be achieved due to the large pore size and volume. Second, enzymes encapsulated into the cavity instead of adsorbing on the MOF surface were less likely to leak from the supports. Third, the size selectivity for specific substrates could be achieved for this typical MOF due to the size limitations.

**FIGURE 7:**
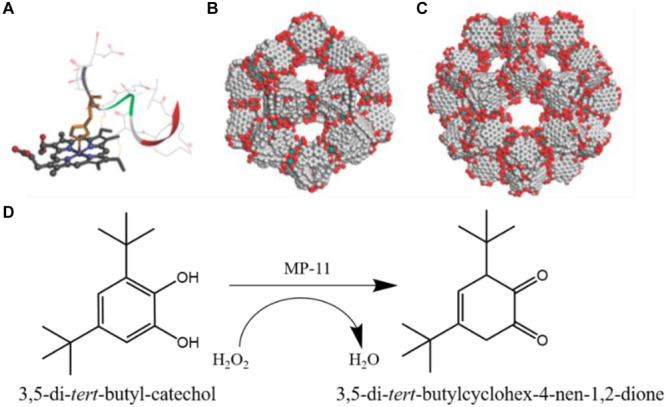
**(A)** Molecular structure of MP-11 (obtained from the solution structure of PDB 1OCD). **(B)** 3.9 nm-diameter cage, and **(C)** 4.7 nm-diameter cage in Tb-mesoMOF. **(D)** Reaction scheme for oxidation of 3,5-di-*tert*-butyl-catechol to *o*-Quinone. Reproduced from Lykourinou et al. (2011) with permission from the American Chemical Society, copyright 2011.

In 2011, Ma et al. first reported encapsulation of the microperoxidase-11 (MP-11) with dimensions of about 3.3 × 1.7 × 1.1 nm (Marques, 2007), into the mesoporous cavities of a terbium-based mesoporous MOF (Tb-mesoMOF, links: triazine-1,3,5-tribenzoate), which showed hierarchical cavities with nanoscopic cages of 3.9 and 4.7 nm in diameter (Figures 7B,C), with pore sizes dominantly distributed around 3.0 and 4.1 nm (Lykourinou et al., 2011). MP-11 that contains an iron-heme group can oxidize a wide range of organic molecules in the presence of hydrogen peroxide (Figure 7A). The N_2_ sorption isotherms analysis indicated the successful entrapment of MP-11 molecules in the pore space of Tb-mesoMOF. The MP-11@Tb-mesoMOF was employed to catalyze the oxidation of 3,5-di-*tert*-butyl-catechol by H_2_O_2_ (Figure 7D). Meanwhile, the mesoporous silica (MCM-41) was also used to immobilize MP-11 as a control group, resulting in a lower loading capacity due to its lower surface area compared to Tb-mesoMOF. The kinetic trace experiments indicated MP-11@Tb-mesoMOF showed a much higher initial rate than that of MP-11@MCM-41during the initial 30 min period. After 25 h, the MP-11@Tb-mesoMOF achieved an improved activity with a final conversion of 48.7%, while free MP-11 and MP-11@MCM-41 only obtained the final conversion of 12.3 and 17.0%, respectively. This outperformance was attributed to the confinement effect of Tb-mesoMOF, which prevented MP-11 from leakage and aggregation. The subsequent cycle experiments also demonstrated that the MP-11 could be better stabilized by the Tb-mesoMOF than MCM-41.

Furthermore, myoglobin (Mb) was also immobilized to extend the application of this Tb-mesoMOF by the same group (Chen et al., 2012a). They demonstrated that the Mb@Tb-mesoMOF had the size selectivity to substrates with different dimensions, such as ABTS (size: 1.01 × 1.73 nm) and 1,2,3-trihydroxybenzene (THB) (size: 0.57 × 0.58 nm). The substrate THB with size-matched to the pore of MOF could easily access the active centers of Mb, resulting in the superior initial rate. Whereas, for the substrate ABTS, which was isolated from the active centers of Mb by pores of MOF, resulted in the very low initial rate. These results indicated that mesoporous MOFs could serve as a novel matrix to immobilize enzyme for applications in size-selective catalysis.

Subsequently, Yaghi et al. developed a strategy to expand the pore aperture of MOF-74. A series of large pore apertures MOFs, ranging from 1.4 to 9.8 nm have been synthesized by systematically expanding the MOF-74 structure (Deng et al., 2012). All these MOF-74 series showed the robust architectures with non-interpenetrating structure, these characteristic with various large pore apertures held potential for the pore entrapment of vitamin B_12_ and green fluorescent protein (GFP).

**FIGURE 8:**
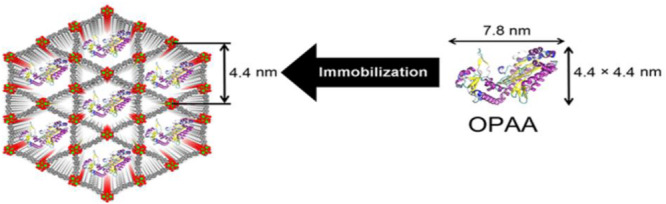
Schematic representation of immobilization of OPAA in the mesoporous channels of NU-1003. Reproduced from Li et al. (2016c) with permission from the American Chemical Society, copyright 2016.

Farha et al. reported a novel application for immobilization of organophosphorus acid anhydrolase (OPAA) with a water-stable Zr-based MOF (NU-1003), featuring the largest mesoporous aperture among Zr-based MOFs (Figure 8; Li et al., 2016c). A size effect of enzyme-MOF composites on their enzymatic activities toward the hydrolysis of nerve agents such as diisopropyl fluorophosphate (DFP), and Soman (GD) was observed. As a result, the OPAA@NU-1003 with a size of 300 nm achieved 3 times faster initial reaction rate than that of free OPAA for GD hydrolysis, which indicated that OPAA@NU-1003-300nm could efficiently deactivate or defluorinated the nerve agents such as DFP and GD.

## Discussion

Metal-organic frameworks have emerged as the comprehensive platform for enzyme immobilization or the synthesis of other hybrid materials since the crystalline structure, pore volume, physical and chemical properties of MOFs can be easily modulated owing to an extensive choice of possible organic linkers and inorganic building units. An obvious advantage of enzyme-MOF composites is their reusability and recyclability, which greatly reduces costs. Besides, a magnetic-MOF that is more convenient for recycling was developed by introducing magnetic moieties to allow magnetic separation from reaction mixtures (Huo et al., 2015; Falcaro et al., 2016). Free enzymes tend to be deactivated when they are exposed to high temperatures, extreme pH, organic solvents, or trypsin digestion, because these external stresses could cause conformational changes of enzymes, resulting in the loss of enzymatic activity. MOFs can provide a microenvironment for immobilized enzymes, which minimizes the loss of enzyme conformation against denaturation conditions. As for enzymatic activity, the enhanced catalytic activity of MOF immobilized enzyme could be achieved with a properly designed matrix or modification of enzymes. For instance, a higher substrate concentration in the vicinity of enzymes would contribute to the enhancement of enzymatic activity, when the adsorption and desorption of substrates and products on supports are well balanced (Zhang et al., 2015). This local enrichment of substrate, also known as the positive partition effect, provides possibilities for MOFs to be served as nanoreactors, which apparently reduces the K_*m*_, and gives an increased apparent enzymatic activity.

Another reason for the enhanced enzymatic activity could be ascribed to highly efficient single enzymes confined by MOF cavities or the functional groups on MOF backbones involved in the catalytic processes (Drout et al., 2019). Additionally, the size of the pore openings may allow MOFs to gain size selectivity. For instance, the peroxidase activity of myoglobin (MB) immobilized in the Tb-mesoMOF was employed for the oxidation of two substrates (ABTS and THB) with different molecular dimensions in the presence of H_2_O_2_ (Chen et al., 2012a). The results indicated that the larger ABTS could not traverse the pores of Tb-mesoMOF, which resulted in very low reactant conversion. In contrast, the composites exhibited a remarkably improved conversion toward the smaller THB.

Similarly, Huo et al. (2015) employed agarose hydrogel droplets stabilized with UiO-66 and Fe_3_O_4_ nanoparticles as the template to synthesize a hierarchically structured ZIF-8 shell. These ZIF-8 shell microcapsules were employed to immobilize the *Candida antarctica* lipase B for transesterification reactions. A size selectivity was also observed for the ZIF-8 shell microcapsules. A complete reactant conversion was observed using a pair of small substrates (1-butanol and vinyl acetate), whereas, only a very little reactant conversion (7.5%) was observed for a pair of large substrates (3-(4-hydroxyphenyl) propan-1-ol and vinyl laurate). This size selectivity could be attributed to the physical barrier imposed by the microporous ZIF-8.

## Applications

**FIGURE 9:**
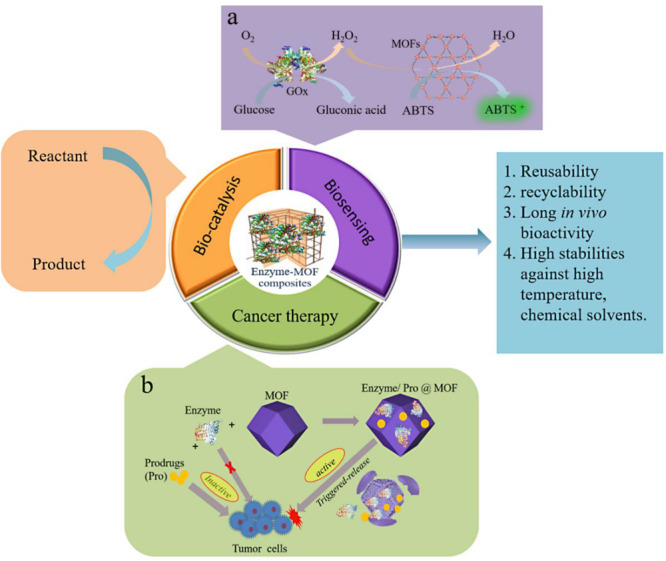
Applications of enzyme-MOF composites. **(a)** Schematic illustration of cascade reaction based on GOx and HRP and its application in bio-sensing. **(b)** Schematic illustration of enzyme/pro@MOF and its application in cancer therapy.

Using MOFs to immobilize enzymes can provide enzymes with excellent reusability, recyclability and long in vivo bio-activity, as well as stabilities against harsh conditions. The applications of MOF bio-composites are extensively and fast developing to different fields including bio-sensing, bio-catalysis, cancer therapy, etc., Figure 9 shows the applications of enzyme-MOF composites. This section mainly focuses on the current progress of enzyme-MOF composites in these fields.

### Bio-Sensing

Recently, bio-sensing and detection based on immobilized enzymes have drawn great attention in many fields such as glucose detection, clinical diagnosis, food monitoring and quality inspection (Patra et al., 2015). For these areas, two basic requirements must be met. One is to respond quickly and accurately at the monitoring site. The other is to continue to function and remain stable in the monitoring environment. Due to the high efficiency and high selectivity of enzymes, the first requirement is not difficult to achieve. However, for the second requirement, the industrial application of enzymes is often limited by their poor stability, lack of reusability and are difficult to recycle (Bornscheuer et al., 2012). Owing to the regulated structure and function, MOFs have become promising candidates to immobilize enzymes in these fields (Feng et al., 2015; Li et al., 2016a; Zhang et al., 2017).

Wang et al. (2017) have reported the co-immobilization of NiPd hollow nanoparticles and GOx on ZIF-8 via an *in situ* synthesis method. The as-prepared GOx@ZIF-8(NiPd) composites showed a nanoflower structure and possessed both glucose oxidase activity and peroxidase-like activity, which could be used as the electrochemical and colorimetric glucose sensor via a tandem catalysis reaction. The results suggested that the absorbance of 2,3-diaminophenazinc at 505 nm showed a good linear relationship to glucose concentrations ranging from 10 to 300 μM, resulting in a LOD of 9.2 μM. And a relative standard deviation of 0.8% was achieved after 16 successive determinations with a wider range (0.1∼1.7 mM) of a linear relationship between the current and glucose concentration, which indicated the good operational stability and expanded the applications of this glucose bio-sensor.

Liu et al. (2018) reported the mimic multienzyme systems for glucose bio-sensor by immobilizing GOx to the hierarchically porous biomimetic Zr-based MOF HP-PCN-224(Fe) (links: tetrakis(4-carboxyphenyl) porphyrin). The hierarchically porous structure of HP-PCN-224(Fe) was prepared through a modulator-induced defect-formation strategy, which introduced dodecanoic acid (DA) as the modulator or surfactant, and reduced the ligand amount to form defects during the MOFs synthesis process, then followed by the hydrochloric acid treatment to remove DA. The DA amount had a great effect on the mesopores formation and the morphology of MOFs. When the molar ratio of DA/ZrCl_4_ was 125, HP-PCN-224(Fe) showed the largest enzyme loading capacity (192.6 mg/g). The intrinsic peroxidase-like activity of HP-PCN-224(Fe), owing to its metalloporphyrin structure, was then employed for the cascade reaction in one step for glucose detection (Figure 9a). A good linear relationship between glucose concentrations ranging from 5 to 300 μM and the absorption value of ABTS^+^ at 420 nm were obtained, with a detection limit of 0.87 μM, which was much lower than other glucose bio-sensors (Hou et al., 2015; Wang et al., 2017). In addition, the universality of HP-PCN-224(Fe) was demonstrated to show the good feasibility for uric acid detection with a detection limit of 1.8 μM, by immobilizing uricase on HP-PCN-224(Fe).

### Bio-Catalysis

Enzymes as prominent natural catalysts with excellent catalytic activity and high selectivity possess great potentials in the manufacture of fine chemicals and pharmaceuticals. In biological catalysis, the function of the enzyme is precisely tuned for highly specific transformations and these transformations proceed in sequences as cascade reactions, which may be involved with multiple enzymes, coenzymes, reactants, and products. This section emphasizes the immobilization of well-understood enzymes with MOFs for applications in bio-catalysis.

**FIGURE 10:**
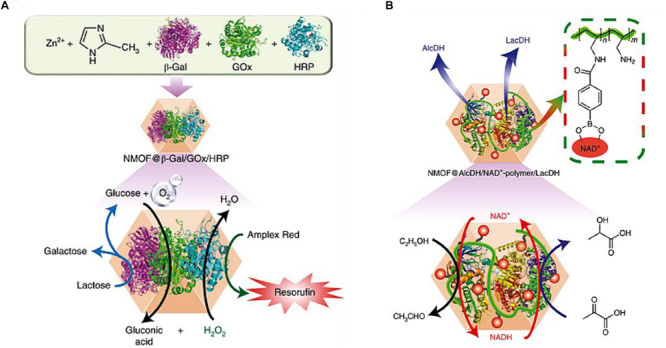
**(A)** Top: composition of the three-enzyme–ZIF-8 NMOF hybrid. Bottom: scheme of the three-enzyme cascades involving β-Gal hydrolysis of lactose and subsequent GOx-catalyzed oxidation of glucose, followed by HRP-catalyzed oxidation of Amplex Red to resorufin by H_2_O_2_. **(B)** Top: composition of the bio-catalytic constituents AlcDH, LacDH and the NAD^+^–polymer in the ZIF-8 NMOF hybrid nanoparticles. Bottom: scheme of the coupled NAD^+^–polymer mediated AlcDH/LacDH-catalyzed reduction of pyruvic acid to lactic acid by the bio-catalytic NMOF hybrid assembly. Reproduced from Chen et al. (2018b) with permission from Nature Publishing Group, copyright 2018.

Inspired by natural bio-catalytic transformations in cells, Chen et al. (2018b) reported complicated bio-catalytic cascades driven by multienzyme in ZIF-8 nanoparticles, which worked as nanoreactors for the concerted reactions of two or three enzymes, as well as cofactor components. As for multienzyme mediated cascade reactions, the intercommunication between enzymes is of paramount importance for the efficiency of reactions. Therefore, ZIF-8 could be qualified as an excellent nanoreactor for transporting the product of one enzyme to the subsequent enzyme, resulting in the high local concentrations of substrates for enzymes. The integration of two enzymes (GOx and HRP) and three enzymes (β-galactosidase (β-Gal), GOx and HRP) (Figure 10A) in ZIF-8 displayed 7.5-fold and 5.3-fold enhanced activity of catalytic cascades, respectively, compared with the homogeneous diffusional mixture of free multienzyme. The improved catalytic performance of cascades could be ascribed to the facilitation of mass transfer that products generated by one enzyme could be utilized by another bio-catalyst. The efficient intercommunication of two β-nicotinamide adenine dinucleotide hydrate (NAD^+^) dependent enzymes (alcohol/lactate dehydrogenase) combined with NAD^+^-functionalized polymer for cascade reactions was also integrated (Figure 10B), which achieved a 4.7-fold improved reduction of pyruvic acid to lactic acid compared with the homogeneous bio-catalytic assembly. These results indicated that the porous structure of MOFs enabled the efficient exchange of substrates and products between the bulk solution and reaction sites.

He et al. (2016) developed a kinetic resolution reactor by encapsulating a thermophilic lipase into the ZIF-8. A simple solid approach by grinding lipase with MOF precursors in trace ethanol was employed to synthesize the lipase@ZIF-8 composites, which was used for the kinetic resolution of (*R, S*)-2-octanol through transesterification. They suggested that lipase@ZIF-8 held the selectivity in (*R, S*)-2-octanol mixture, and preferably catalyzed the *R*-form. Superior enzymatic activity of lipase@ZIF-8 in a non-aqueous medium was observed. As a result, lipase@ZIF-8 showed advantageous reusability in the kinetic resolution of *sec*-alcohols with stable *E* values ranging from 8.1 to 11.5 during 10 cycles at room temperature. Owing to the protection of MOFs, the immobilized enzyme exhibited enhanced resistance against trypsin. These results indicated that enzyme-MOF could be potentially beneficial for the cost-effective synthesis of chiral compounds via bio-catalysis.

### Cancer Therapy

Using functional proteins, DNA/RNA for cancer treatment is of importance in both clinical and preclinical studies. Usually, chemotherapeutics tends to show the weak targeting effect or cause toxic effects on normal cells during cancer treatment. One strategy to get around this problem is to employ nontoxic prodrugs metabolized by enzymes to generate cytotoxic products in a tumor microenvironment (Giang et al., 2014; Lin et al., 2018). Nevertheless, enzymes in cells is in very low levels, which leads to the poor selectivity and unsatisfactory results for this enzyme-activated prodrug therapies. An ideal method is to deliver exogenous enzymes to tumor cells. However, enzymes tend to suffer from the internal environment during the delivery process. This problem can be solved by the immobilization of enzymes with MOFs, which emerges as excellent matrixes to deliver enzymes to the cancer cells due to their low toxicity, high encapsulation efficiency, high loading capacity and good bio-compatibility, etc.

As well known, the rapid proliferation of tumor cells requires a large amount of glucose. GOx can catalyze glucose to produce glucuronic acid and hydrogen peroxide, which may produce the hydroxyl radical through Fenton reaction (Huo et al., 2017), or oxidize arginine to generate NO, resulting in the starvation synergistic cancer therapy (Fan et al., 2017). This process could also be enhanced by using a cytostatic environment and even toxicity of the drugs (Zhang R. et al., 2018; Zhou et al., 2018; Chu et al., 2019). Zhang L. et al. (2018) constructed a biomimetic nanoreactor by encapsulating prodrug tirapazamine (TPZ) and GOx in an erythrocyte membrane cloaked MOF nanoparticle (TGZ@eM). The acidic environment caused by lysosome in cells could disintegrate the TGZ@eM to release GOx and TPZ. The releasing GOx consumed exogenous glucose and oxygen to starve cancer cells, and thus the hypoxic environment caused TPZ to be more toxic and inhibit the growth and reproduction of cancer cells (Figure 9b). As a result, a tumor growth inhibition (TGI) rate of 97.6% was obtained when the TGZ@eM nanoreactor was administered intravenously, which afforded the most satisfactory therapeutic outcomes. Coincidentally, Chen et al. (2018c) simultaneously co-immobilized the GOx and insulin or anti-vascular endothelial growth factor aptamer (VEGF aptamer) in ZIF-8 to construct a two-step sense-and-treat system for macular diseases. In these systems, the VEGF aptamer could be controlled release mediated by the GOx-catalyzed aerobic oxidation glucose, which could inhibit the VEGF-induced generation of blood vessels and the high glucose concentrations in cancer cells, and held potentials for other disease treatment.

**FIGURE 11:**
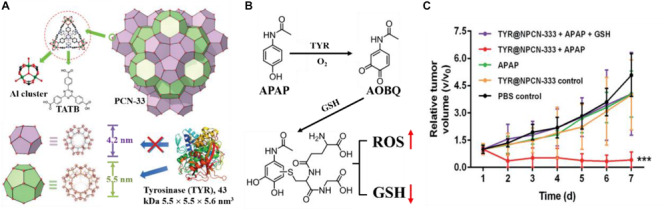
**(A)** Top: structure of PCN-333 composed of aluminum trimeric cluster and 4,4′,4″-s-triazine-2,4,6-triyl-tribenzoic acid (TATB) ligand. Bottom: two types of mesoporous cavities in PCN-33 (purple: 4.2 nm, green: 5.5 nm), TYR with size of 5.5 × 5.5 × 5.6 nm^3^, which can only be accommodated in the 5.5 nm cage. **(B)** Nontoxic prodrug APAP oxidation is catalyzed by TYR to yield cytotoxic AOBQ, which induces ROS generation and GSH depletion. **(C)** Time-dependent relative tumor growth upon different treatments. Reproduced from Lian et al. (2018) with permission from Wiley Online Library, copyright 2018.

Lian et al. (2018) developed an approach to construct the prodrug activation mediated by exogenous enzymes for cancer therapy with better selectivity and less toxicity. Tyrosinase (TYR) was chosen as the activating enzyme and loaded in the PCN-333 nanoparticles (NPCN-333) (Figure 11A). The obtained TYR@NPCN-333 could activate the prodrug of paracetamol (APAP) in cancer cell in a prolonged way. The cytotoxicity arose from 4-acetamido-*o*-benzoquinone (AOBQ), which was the enzymatic transformation product of nontoxic prodrug APAP, and from subsequently inductive generation of reactive oxygen species (ROS) and glutathione (GSH) depletion (Figure 11B). As a result, a 2.5-time regression of tumor volume was achieved after the treatment with TYR@NPCN-333 and APAP (Figure 11C). Chen et al. (2018a) developed the protein-encapsulated MOF composites as a novel platform for intracellular delivery of native active proteins, and demonstrated that protein-encapsulated MOF composites could serve as a generally applicable platform for protein delivery and protein-based theranostics.

Briefly, there are mainly two ways of applying of enzyme-MOF in cancer treatment: (i) enzymes are released from enzyme-MOFs in the tumor microenvironment, then directly affect or synergistically work together with prodrugs to the tumor cells; (ii) enzymes act on cancer cells by changing the tumor microenvironment or activating prodrugs.

## Conclusion and Prospects

Metal-organic frameworks are emerging as viable host matrixes for enzyme immobilization. The immobilization strategies such as surface immobilization, *in situ* approach, covalent linkage and pore entrapment have been developed. All these approaches allow the immobilized enzyme to be reusable and recyclable. The use of MOFs to immobilize enzymes is gaining increasing attention, however, related researches are still in the preliminary stages. Many aspects need to be further improved. For instance, enzymes adsorbed on the surface of MOFs through weak interactions tend to leak from the support during the recycled usage. Though this problem can be addressed to a great extent by the covalent linkage approach, where the firm interaction between immobilized enzyme and support is formed. Both approaches result in immobilized enzymes exposed to the environment, which causes enzymes vulnerable to harsh conditions or digestion by proteases. The pore entrapment approach allows enzymes to exhibit excellent reusability and resistance against denaturation agents and against harsh conditions, when they are entrapped into the pores or cages of the mesoporous MOFs. Nevertheless, pore apertures of the mesoporous MOFs should be suitable for the size of enzyme in this fashion. Reasonable design for the MOFs to immobilize enzymes varied vastly in shape and size remains a challenge, and the synthesis of mesoporous MOFs fraught with difficulties due to the undesirable interpenetrating structures. The *in situ* synthesis could address many of the problems mentioned above, yet it can be employed only under mild synthetic conditions such as in an aqueous solution. Usually, ZIFs serve as the most common candidates for this approach. However, further applications of enzyme-ZIFs composites are limited by the mass transfer of substrates and products due to the narrow micropores of ZIFs. Therefore, approaches for enzyme immobilization should depend on the specific enzyme-MOF couple and practical situation of the catalytic process, and the novel approach needs to be developed urgently.

Although enzyme-MOFs composites have been widely reported to be used in the field of bio-sensing and detection, bio-catalysis and recent cancer therapy, employing the composites in industrial or biomedical conditions are rarely reported. This is mainly hampered by deficient chemical stability, especially in water, and difficulty in regulating the structure and aperture of MOFs. Overcoming these shortcomings helps to facilitate the immobilization processes and to implement them in practical production. It is worth mentioning that only a few researches focus on the facile synthesis or post-synthetic modification amenability of MOFs, which is crucial for the scale-up of laboratory synthesis into mass production. In addition, the host-guest interaction has been investigated by comprehensive measurements, such as fluorescence, UV-vis, circular dichroism, and dynamic light scattering (Anand et al., 2014; Xu et al., 2017), yet there is still a deficiency of insight into the interactions between enzyme and MOF matrixes, especially the diffusion mechanism of enzymes into the cages or pores of mesoporous MOFs.

The synergistic effect between MOFs and enzymes can be achieved by the rational design of MOFs combined with the functionality of enzymes. For instance, the enantioselectivity of enzymes could be enhanced by introducing asymmetric groups to MOFs. And the modification of MOFs with hydrophilic groups could facilitate the interaction of hydrophobic enzymes with hydrophilic substrates, giving rise to a higher reactant conversion and product yield. A biomimetic MOF could also be combined with enzymes to construct a mimic multi-enzyme system for tandem catalysis.

Finally, theoretical simulation is also important since it provides guidelines for the tuning of MOF pores or cages, and for size matching between enzymes and MOF cavities. Thus, the loading optimization, dispersion and orientation of immobilized enzymes could be achieved with a rational theoretical calculation. The enzyme-MOF composites could contribute to the development of the next wave of applied bio-catalysis, once the set of design rules combined with theoretical simulation have been established.

## Author Contributions

The author sequence is based on the contribution to the study without controversy. All authors contributed to the article and approved the submitted version.

## Conflict of Interest

The authors declare that the research was conducted in the absence of any commercial or financial relationships that could be construed as a potential conflict of interest.
